# Transfer Learning-Based Multi-Sensor Approach for Predicting Keyhole Depth in Laser Welding of 780DP Steel

**DOI:** 10.3390/ma18173961

**Published:** 2025-08-24

**Authors:** Byeong-Jin Kim, Young-Min Kim, Cheolhee Kim

**Affiliations:** 1 Flexible Manufacturing R&D Department, Korea Institute of Industrial Technology, Incheon 21999, Republic of Korea; bjkim0403@kitech.re.kr (B.-J.K.); ymkim77@kitech.re.kr (Y.-M.K.); 2 Department of Mechanical Convergence Engineering, Hanyang University, Seoul 04763, Republic of Korea; 3 Department of Mechanical and Materials Engineering, Portland State University, Portland, OR 97229, USA

**Keywords:** laser welding, penetration-depth estimation, deep learning, transfer learning, multi-sensor model, CNN

## Abstract

Penetration depth is a critical factor determining joint strength in butt welding; however, it is difficult to monitor in keyhole-mode laser welding due to the dynamic nature of the keyhole. Recently, optical coherence tomography (OCT) has been introduced for real-time keyhole depth measurement, though accurate results require meticulous calibration. In this study, deep learning-based models were developed to estimate penetration depth in laser welding of 780 dual-phase (DP) steel. The models utilized coaxial weld pool images and spectrometer signals as inputs, with OCT signals serving as the output reference. Both uni-sensor models (based on coaxial pool images) and multi-sensor models (incorporating spectrometer data) were developed using transfer learning techniques based on pre-trained convolutional neural network (CNN) architectures including MobileNetV2, ResNet50V2, EfficientNetB3, and Xception. The coefficients of determination values (R^2^) of the uni-sensor CNN transfer learning models without fine-tuning ranged from 0.502 to 0.681, and the mean absolute errors (MAEs) ranged from 0.152 mm to 0.196 mm. In the fine-tuning models, R^2^ decreased by more than 17%, and MAE increased by more than 11% compared to the previous models without fine-tuning. In addition, in the multi-sensor model, R^2^ ranged from 0.900 to 0.956, and MAE ranged from 0.058 mm to 0.086 mm, showing better performance than uni-sensor CNN transfer learning models. This study demonstrated the potential of using CNN transfer learning models for predicting penetration depth in laser welding of 780DP steel.

## 1. Introduction

Laser welding has become an essential joining technique in modern manufacturing due to its high energy density, narrow heat-affected zone (HAZ), and high processing speed [1]. This technology is particularly prevalent in the automotive industry, where it is widely used for joining advanced high-strength steels (AHSS) [2,3,4], such as dual-phase (DP) steels that offer an optimal balance between strength and ductility [2,5,6]. These materials enable the production of lightweight vehicle structures with improved crashworthiness [7,8]. To ensure the mechanical integrity of welded assemblies, it is critical to precisely control and monitor welding quality parameters—among which penetration depth is especially important [9,10], as it directly impacts the structural soundness and load-bearing capacity of the joint. However, in keyhole-mode laser welding, the dynamic and unstable nature of the keyhole significantly complicates real-time depth monitoring [11,12,13]. Traditional evaluation methods, such as destructive testing or radiographic inspection, though reliable, are labor-intensive, costly, and unsuitable for inline inspection in high-throughput industrial environments [14,15]. To overcome these limitations, optical coherence tomography (OCT) [16,17] has been introduced as a non-destructive, high-resolution technique capable of capturing the internal geometry of the keyhole during welding operations [18,19]. While OCT offers distinct advantages in mapping the keyhole morphology, its practical application is often hindered by noise susceptibility, signal degradation, and alignment sensitivity due to process-induced disturbances such as spatter [20] or keyhole fluctuations [21,22]. In response to these challenges, recent advances in deep learning have opened new possibilities for weld quality prediction [23,24]. In particular, convolutional neural networks (CNNs) [25,26], when combined with transfer learning strategies, have shown substantial promise in extracting complex features from high-dimensional sensor data—such as weld pool images and spectral signals—for accurate, real-time estimation of weld characteristics, including penetration depth [27,28,29,30,31].

This study presents a multi-sensor deep learning framework for predicting penetration depth during laser welding of 780DP steel. The proposed model employs coaxial camera images and spectrometer signals as inputs and uses calibrated OCT data as the ground truth. A comparative analysis is conducted using CNN-based transfer learning models—including MobileNetV2 [32,33], ResNet50V2 [34], EfficientNetB3 [35], and Xception [36]—under both uni-sensor and multi-sensor configurations. The results demonstrate that the multi-sensor approach significantly enhances prediction accuracy, highlighting its potential for real-time penetration depth estimation in industrial laser welding applications.

## 2. Experiments

**Figure 1:**
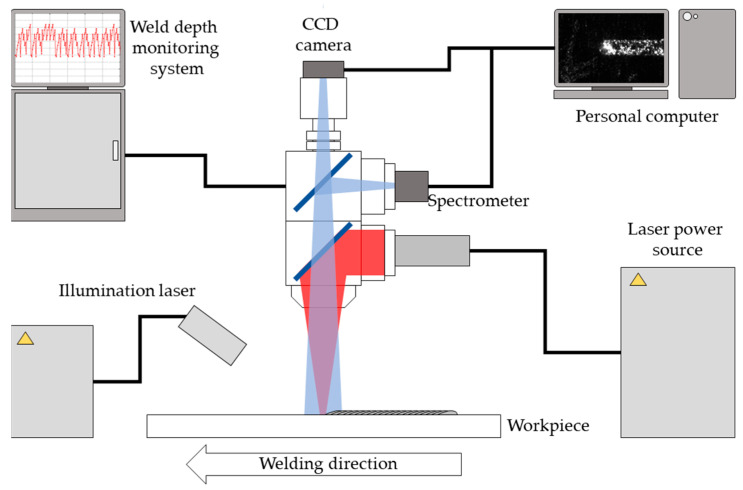
Schematic diagram of data collection system.

The base material used in this study was 780 dual-phase (DP) steel with a thickness of 3.5 mm. Bead-on-plate (BOP) welding tests were conducted to evaluate penetration behavior. The chemical composition and mechanical properties of the steel are summarized in Table 1 and Table 2. Steel specimens were prepared as rectangular sheets measuring 50 mm in width and 150 mm in length, with a weld length of 100 mm. The laser system employed a fiber laser (YLS-6000, IPG Photonics, USA) with a beam quality of 2 mm·mrad and a maximum output power of 6 kW. Laser energy was delivered to the welding head through an optical fiber with a core diameter of 200 μm. The laser welding head was equipped with focusing optics (D30 Wobble, IPG Photonics, USA), an illumination laser system, an OCT sensor (LDD-700, IPG Photonics, USA), a charge-coupled device (CCD) camera (UI-3140CP Rev. 2, iDS, Germany), and a spectrometer (HR4000, Ocean Optics, USA) (Figure 1). The focal length of the optics was 200 mm, and the laser beam diameter at the specimen surface was approximately 270 μm. An illumination laser operating at a wavelength of 980 nm was used, with an output power of 100 W and an incident angle of 40° relative to the specimen’s surface. The OCT sensor, operating at a sampling frequency of 135 kHz, was used to monitor the depth of the keyhole in real time. Since the OCT system measures keyhole depth directly, a calibration procedure was conducted to correlate the OCT signal with actual penetration depth, enabling its use as the target output for model training. Images of the weld zone were captured by the CCD camera through a 980 ± 5 nm bandpass filter at a frame rate of 500 Hz. The spectrometer detected signals in the wavelength range of 200 to 1100 nm, with a sampling rate of 100 Hz and an optical resolution of 0.47 nm at half spatial resolution. Both the CCD camera and the spectrometer were optically aligned with the welding head using a dichroic mirror.

Table 3 provides details of the welding parameters used in the experiment. The welding speed was varied from 3 to 7 m/min in 1 m/min increments, and laser power was adjusted accordingly to produce welds with different penetration depths. Data were collected from the CCD camera, OCT sensor, and spectrometer at their respective sampling rates, and were synchronized to 100 Hz and 500 Hz datasets. The first and last 0.3 s of each weld sequence were excluded from model training to eliminate start/end edge effects.

## 3. Data Preprocessing and Models

### 3.1. Penetration Depth Calibration

**Figure 2:**
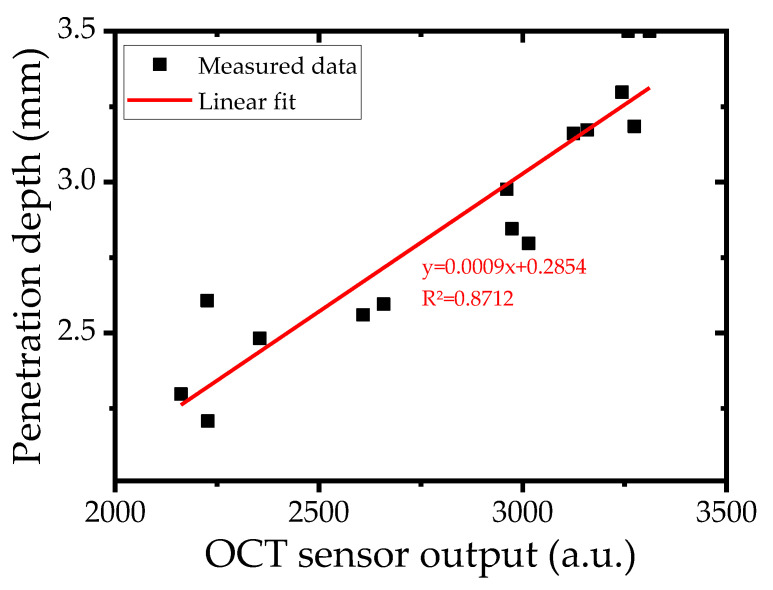
Relationship between OCT signal and penetration depth.

The OCT signal was utilized to estimate the laser weld penetration depth by serving as a proxy for keyhole depth. A calibration process was conducted to establish the correlation between OCT measurements and the actual penetration depth. For this purpose, specimens welded under 15 discrete welding conditions were randomly sectioned to obtain coupons, and the penetration depth was measured through metallographic examination. The mean values of the OCT signals obtained at the corresponding locations and then the measured penetration depths were plotted (Figure 2). A linear regression yielded a coefficient of determination (R^2^) of 0.8712, indicating a strong correlation.

### 3.2. Data Preprocessing

**Figure 3:**
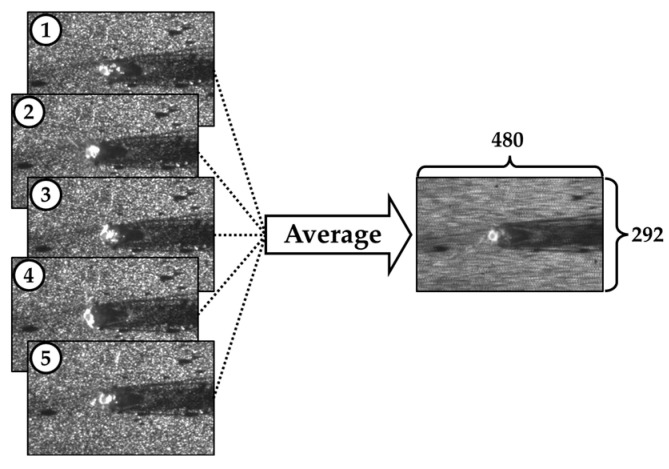
Pretreatment process of image data: downsampling of images using the averaging algorithm.

**Figure 4:**
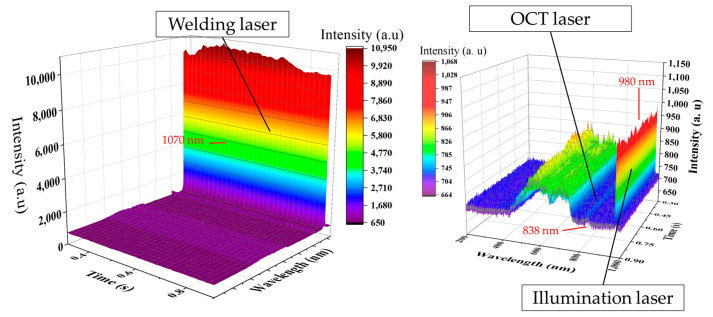
Spectrum intensity of laser welding.

**Figure 5:**
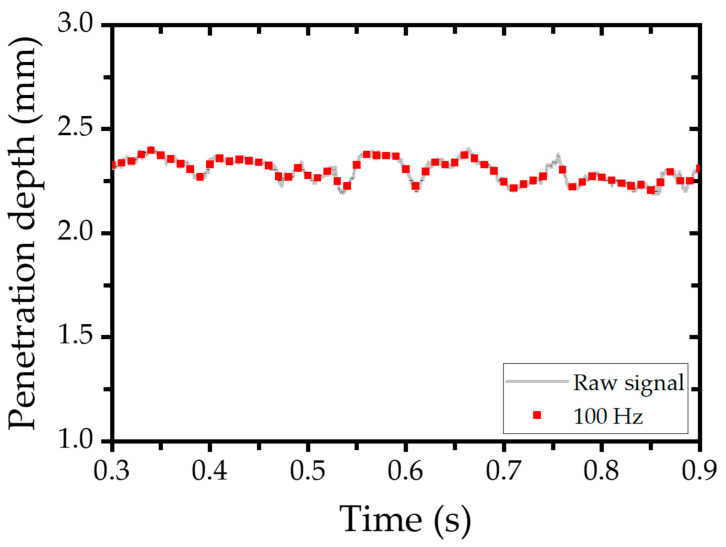
Comparison of raw data and 100 Hz sampled data on OCT signal.

The model inputs—CCD camera images and spectrometer signals—and the target output—penetration depth—were collected at different sampling frequencies. Specifically, data were acquired at 100 Hz and 500 Hz depending on the sensor, and separate models were trained and evaluated based on these two sampling rates. CCD images capturing the weld pool, keyhole, and bead were obtained at 500 Hz using a coaxial camera aligned with the laser optics. The image resolution was 292 × 480 pixels. To align with the 100 Hz sampling rate of other sensors, images were downsampled by averaging every five consecutive frames. Although this resampling process resulted in slightly blurred images, the key structural features remained distinguishable (Figure 3). Spectrometer signals were acquired at a sampling frequency of 100 Hz. For the 100 Hz model, these signals were used directly. For models operating at 500 Hz, spectrometer data were upsampled using the Fourier transform method to match the target frequency. Figure 4 illustrates key spectral features, including peaks at 1070 nm (laser wavelength), 980 nm (illumination laser), and approximately 838 nm (OCT reference). The OCT sensor operated at a high frequency of 135 kHz. To synchronize with the models’ sampling rates, OCT data were averaged over windows of 1350 and 270 samples, yielding effective rates of 100 Hz and 500 Hz, respectively. Figure 5 compares raw OCT data with their downsampled counterpart, demonstrating that essential signal characteristics were preserved despite slight attenuation.

### 3.3. Deep Learning Models

**Figure 6:**
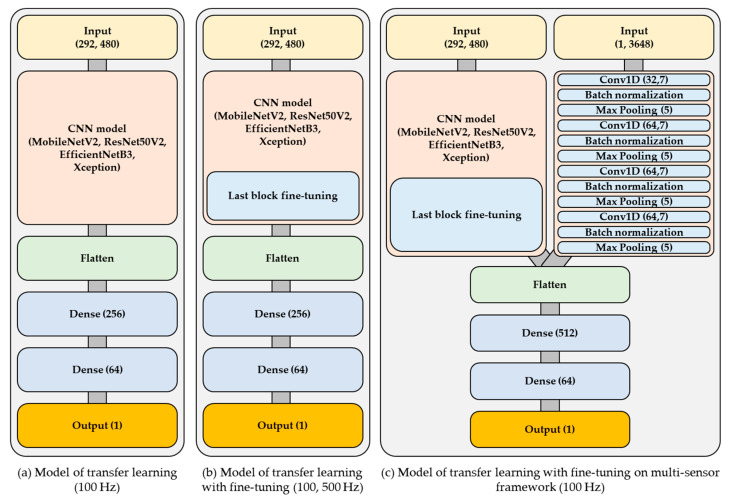
Structure of the models: (**a**) model of transfer learning (100 Hz); (**b**) model of transfer learning with fine-tuning (100, 500 Hz); (**c**) model of transfer learning with fine-tuning on multi-sensor framework (100 Hz).

The deep learning framework included three types of models: (1) a baseline single-sensor transfer learning model, (2) a fine-tuned single-sensor model trained on both 100 Hz and 500 Hz data, and (3) a fine-tuned multi-sensor model designed to enhance prediction of laser weld penetration depth. The single-sensor model was based on a conventional convolutional neural network (CNN) architecture and was trained using CCD camera images sampled at either 100 Hz or 500 Hz. Transfer learning was applied using several pre-trained models, including MobileNetV2, ResNet50V2, EfficientNetB3, and Xception. For the 100 Hz configuration, CCD images downsampled to 100 Hz served as inputs, and the corresponding downsampled penetration depth data were used as outputs (Figure 6a). In the fine-tuned single-sensor model (Figure 6b), the last block of the pre-trained architecture was adjusted during training. Both original 500 Hz CCD images and their 100 Hz downsampled versions were used as inputs, with penetration depth data processed similarly via upsampling or downsampling to match. The multi-sensor model (Figure 6c) extended the fine-tuned single-sensor framework by integrating spectrometer data sampled at 100 Hz as an additional input. This fusion of CCD imagery and spectrometer signals aimed to improve model accuracy through sensor complementarity. All models employed the Rectified Linear Unit (ReLU) as the activation function for both hidden layers and output nodes.

### 3.4. Dataset and Optimization Method

Following data collection and preprocessing, a total of 1769 and 8845 data samples were obtained for the 100 Hz and 500 Hz datasets, respectively. These datasets were randomly split into training, validation, and testing subsets in a 70:15:15 ratio to ensure balanced model evaluation. Model training was conducted using the Adam optimization algorithm. The optimizer was configured with the following hyperparameters: learning rate = 0.001, β_1_ = 0.9, β_2_ = 0.999, and ε = 1 × 10^−8^. Each model was trained over 1000 epochs to ensure convergence.

## 4. Results

**Figure 7:**
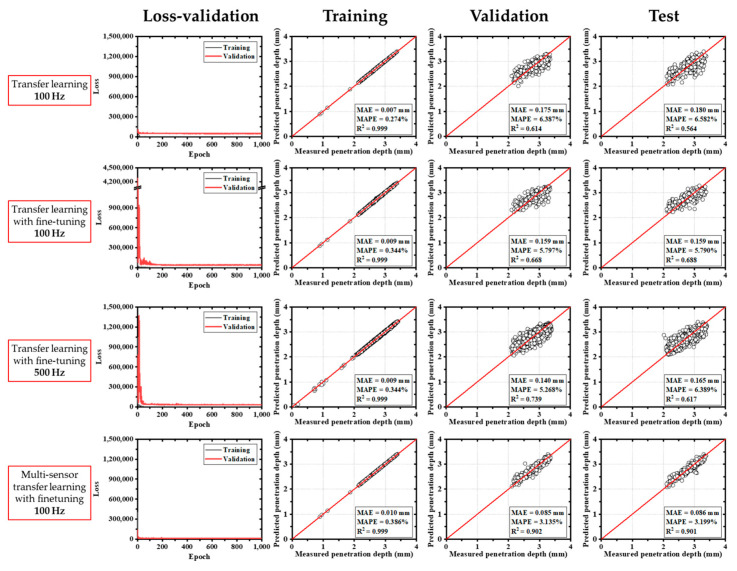
Results of deep learning with transfer learning (MobileNetV2).

The deep learning model based on the MobileNetV2 transfer learning architecture demonstrated rapid convergence of training loss during the initial epochs, as illustrated in Figure 7. Across various input configurations, the model yielded mean absolute error (MAE) values ranging from 0.086 mm to 0.180 mm. In addition to the MAE, the mean absolute percentage error (MAPE) was calculated to represent the prediction error as a percentage of the actual penetration depth, thereby providing a more intuitive assessment of the model’s accuracy. The MAPE is defined as the mean of the absolute differences between the predicted and actual values, normalized by the actual values and expressed as a percentage. Among the evaluated models, the fine-tuned multi-sensor configuration achieved the lowest MAE and the highest coefficient of determination (R^2^ = 0.901), indicating superior prediction accuracy. Although both training and validation datasets showed relatively low errors, the test dataset exhibited increased error and a decline in R^2^, suggesting potential overfitting or limited generalizability. In fine-tuned single-sensor models, a sharp increase in loss was observed during early training, which stabilized after approximately 150 epochs.

**Figure 8:**
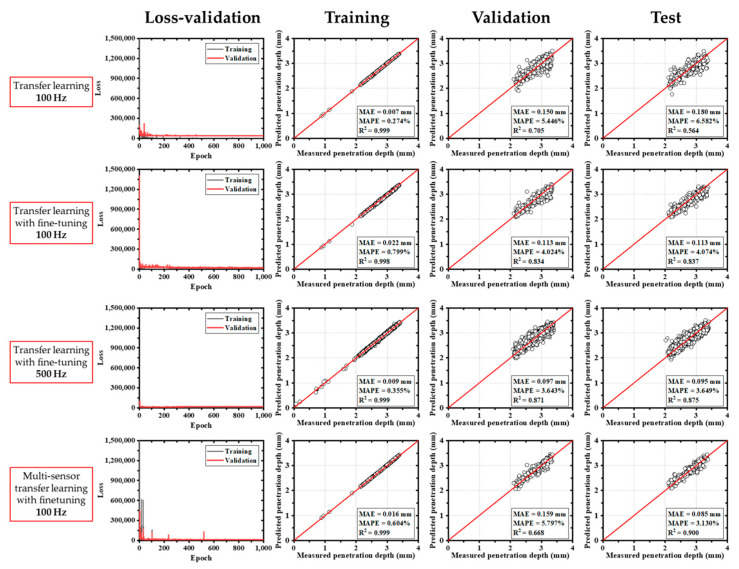
Results of deep learning with transfer learning (ResNet50V2).

In comparison, models based on the ResNet50V2 architecture exhibited a more gradual reduction in training loss, reaching stable convergence after around 200 epochs. As shown in Figure 8, the ResNet50V2 model maintained relatively stable loss from the beginning, even when fine-tuning was applied. For this model, MAEs ranged from 0.085 mm to 0.180 mm, and R^2^ values ranged from 0.564 to 0.900.

**Figure 9:**
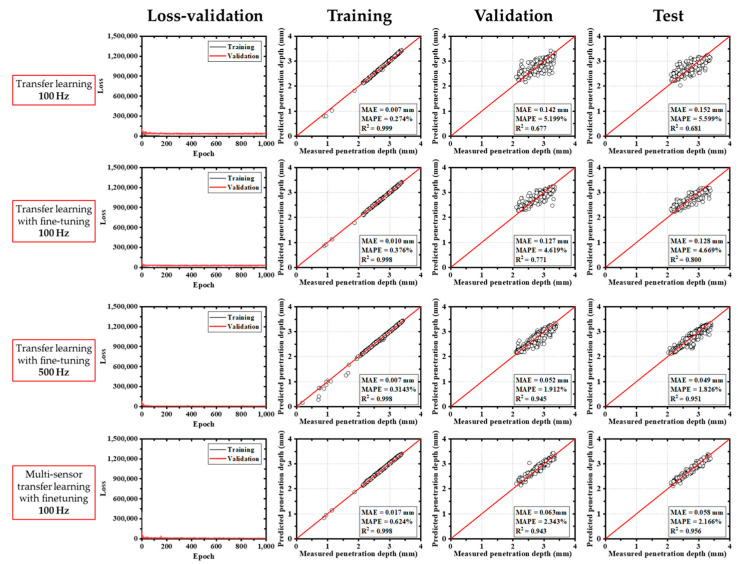
Results of deep learning with transfer learning (EfficientNetB3).

The EfficientNetB3-based models produced the lowest training errors overall. The MAEs ranged from 0.049 mm to 0.152 mm, and the R^2^ values spanned 0.681 to 0.956, as shown in Figure 9. This result demonstrates that EfficientNetB3 offered improved predictive performance relative to other architectures.

**Figure 10:**
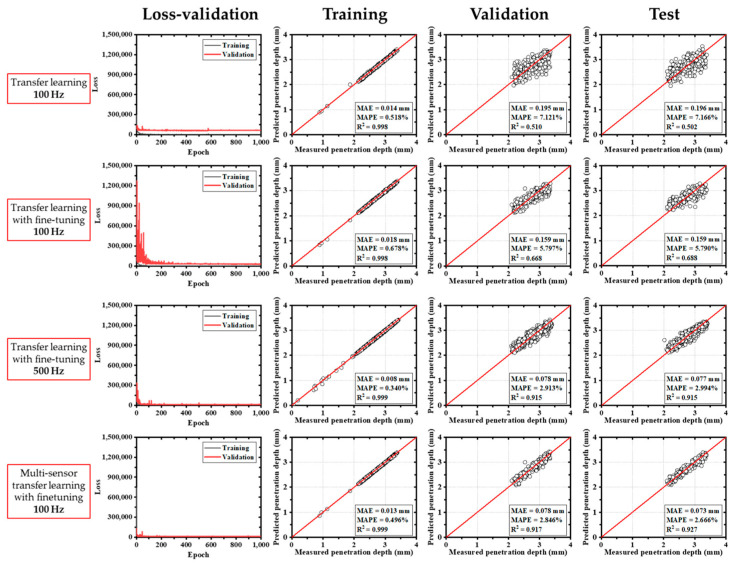
Results of deep learning with transfer learning (Xception).

Similarly, models using the Xception architecture showed consistent convergence behavior after a certain number of epochs (Figure 10). These models yielded MAEs between 0.073 mm and 0.196 mm, and R^2^ values between 0.502 and 0.927.

**Figure 11:**
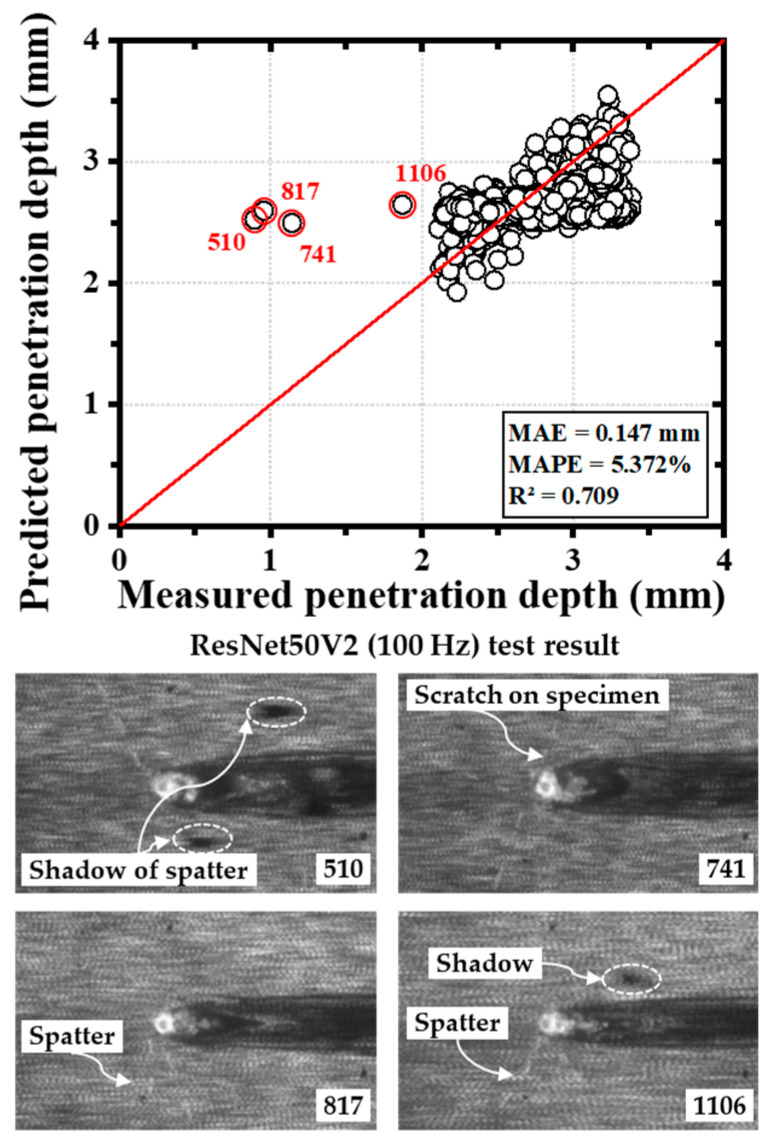
Outliers occurring in deep learning model during training.

Outliers were identified during model training. For instance, in the ResNet50V2 model, data points #510, #741, #817, and #1106 deviated significantly from the ideal x = y line in the scatter plots (Figure 11). These anomalies were attributed to imaging artifacts such as spatter, shadows, or scratches that caused inaccuracies in OCT signal acquisition. These errors were addressed during data refinement. Overall, comparative analysis revealed that MobileNetV2 models often achieved the lowest MAEs and highest R^2^ values—up to 0.998 in some training cases.

**Figure 12:**
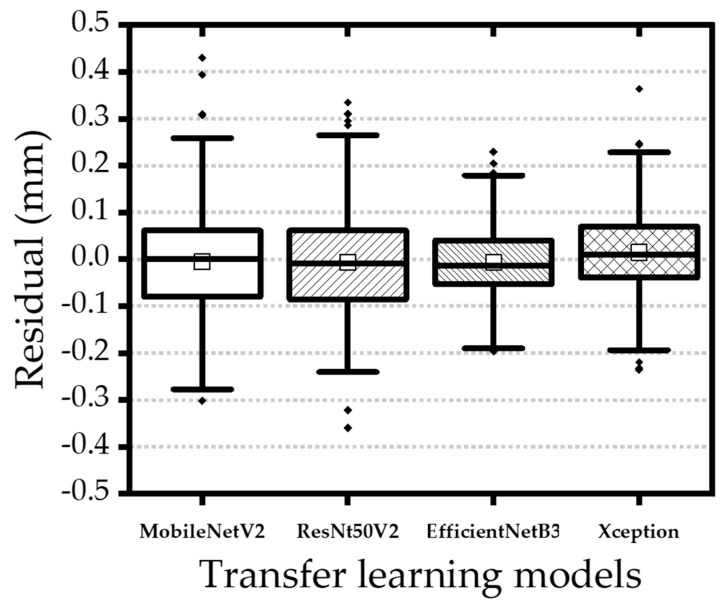
Outliers occurring in deep learning models during training (transfer learning with fine-tuned multi-sensor model, 100 Hz).

**Figure 13:**
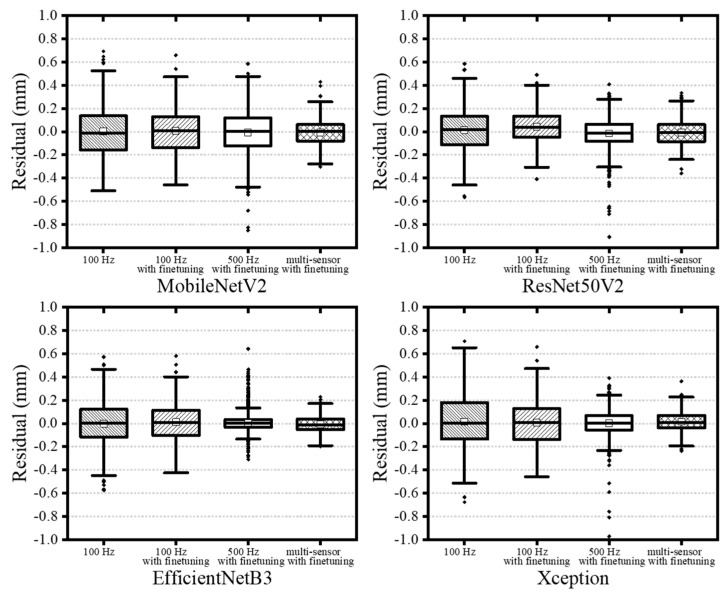
Comparison of test results depending on dataset.

Among all configurations, the single-sensor model trained on 500 Hz CCD data achieved the highest training accuracy (Table 4). However, validation performance was notably lower, with MAEs increasing by factors of 6 to 25 and R^2^ decreasing by 5% to 48% (Table 5), indicating generalization limitations. Despite this, all architectures showed consistent trends between training and validation. The EfficientNetB3 model demonstrated the most robust accuracy, while the multi-sensor model using 100 Hz data produced the most balanced and generalizable performance (Table 6). Figure 12 and Figure 13 present box plots comparing model performance. EfficientNetB3 had the narrowest interquartile range (−0.05347 mm to 0.04045 mm), whereas MobileNetV2 exhibited the widest spread (−0.08005 mm to 0.06178 mm). Although ResNet50V2 presented more frequent outliers, the largest deviations were observed in MobileNetV2 results. In general, multi-sensor models with fine-tuning yielded narrower error distributions, particularly when operating on 100 Hz data. The EfficientNetB3 model demonstrated the most robust accuracy, while the multi-sensor model using 100 Hz data produced the most balanced and generalizable performance. Figure 12 and Figure 13 present box plots comparing model performance. EfficientNetB3 had the narrowest interquartile range (−0.05347 mm to 0.04045 mm), whereas MobileNetV2 exhibited the widest spread (−0.08005 mm to 0.06178 mm). Although ResNet50V2 presented more frequent outliers, the largest deviations were observed in MobileNetV2 results. In general, multi-sensor models with fine-tuning yielded narrower error distributions, particularly when operating on 100 Hz data.

## 5. Discussion

The most accurate model developed in this study achieved a mean absolute error (MAE) of 0.049 mm in predicting penetration depth during laser welding. Given the base material thickness of 3.5 mm, this corresponds to an error margin of approximately 1.4%, underscoring the high precision of the proposed approach. This research explored four distinct transfer learning architectures; however, many additional deep learning models remain to be evaluated in future work. Among the evaluated architectures, EfficientNetB3 consistently achieved the highest prediction accuracy, while the multi-sensor configuration outperformed single-sensor counterparts overall [26]. Although increasing the size of the training dataset generally enhances model accuracy, it also significantly prolongs training time. Therefore, model selection should be informed by the constraints and requirements of specific industrial applications. Additionally, due to varying sampling rates across different sensors, rigorous preprocessing and synchronization are necessary. In this study, the spectrometer signal—despite its relative and sensitive nature—proved effective as a supplementary input for assessing weld quality [29,31]. By averaging every five images into one, the CCD image data were downsampled from 500 Hz to 100 Hz, ensuring compatibility across input datasets without losing relevant information. In some cases, models trained on CCD data sampled at 100 Hz achieved higher prediction accuracy than those trained on 500 Hz data. This improvement can be attributed to the reduction of noise and extraneous fluctuations inherent in higher-frequency image acquisition. Thus, the 100 Hz dataset retained sufficient informational content while mitigating noise effects, leading to enhanced performance in certain model architectures. Furthermore, the high acquisition frequency of the OCT sensor (135 kHz) was instrumental in reducing the effects of keyhole instability, a known challenge in laser welding processes. In certain fine-tuned configurations, a reduction in prediction accuracy was observed. This phenomenon can be attributed to overfitting, where the model became overly specialized to the characteristic weld pool morphology of 780DP steel present in the training dataset. As a result, its ability to generalize to variations in weld pool appearance—caused by process instabilities, sensor noise, or differing visual features—was diminished, leading to reduced performance on unseen data. Most notably, this study demonstrated that deep learning models can accurately predict penetration depth without the need for destructive testing or radiographic inspection, offering a viable alternative for real-time, non-destructive weld quality assessment. It is anticipated that significant changes in surface conditions, such as oxidation, contamination, or increased roughness, could degrade sensor signal quality and reduce prediction accuracy, as evidenced by spatter-induced artifacts that introduced outliers during model training.

Beyond penetration depth prediction, the proposed multi-sensor framework has strong potential to be extended for comprehensive weld quality assessment. Process instabilities in keyhole-mode laser welding—such as spatter generation, porosity formation, and undercut—are often correlated with the same physical phenomena that influence penetration depth. By incorporating additional labeled datasets containing these defect types, the current model architecture could be adapted for multi-target learning, enabling simultaneous prediction of penetration depth and defect occurrence [37]. Moreover, the integration of supplementary sensing modalities, such as high-speed imaging or acoustic emission monitoring, could provide richer process signatures, thereby improving the model’s capability to capture transient anomalies and subtle defect precursors. Such an extended system would contribute to real-time, inline weld quality monitoring, reducing reliance on destructive or radiographic testing and facilitating the implementation of closed-loop process control. Schmoeller et al. (2022) demonstrated an inline OCT-based control system capable of real-time weld depth regulation using closed-loop feedback, suggesting that similar frameworks could be adapted for industrial deployment [38].

While the experimental methodology demonstrated in this study was validated on bead-on-plate laser welding of 780DP steel, its direct applicability to materials with substantially different thermal or mechanical properties, or to markedly different welding conditions, remains to be verified. Consequently, the conclusions drawn here should be interpreted within the scope of the tested material and process parameters. Future research will aim to extend the framework to a broader range of materials and welding setups to assess its generalizability.

## 6. Conclusions

In this study, a deep learning model incorporating transfer learning techniques was developed to predict penetration depth in bead-on-plate (BOP) laser welding of 780DP steel. The model utilized CCD camera images and spectrometer signals as input features, while the OCT signal—correlated with actual weld depth—was used as the output reference. Both single-sensor and multi-sensor configurations were explored using preprocessed datasets.

The following conclusions can be drawn:

(1) A transfer learning-based deep learning model was successfully implemented for predicting weld penetration depth. The best-performing configuration achieved a mean absolute error (MAE) of 0.049 mm and a coefficient of determination (R^2^) of 0.951, corresponding to approximately 1.5% error relative to the material thickness—indicating high prediction accuracy.
(2) CCD imagery and spectrometer signals were found to be effective input features. The use of a bandpass filter and illumination laser enhanced the quality and reliability of the captured images. Additionally, the high-frequency OCT sensor provided robust reference measurements, minimizing keyhole instability and contributing to the model’s strong performance.
(3) The experimental methodology, although based on BOP testing of 780DP steel, shows strong potential for generalization to other steel materials and welding conditions.

This research demonstrates the feasibility of accurate, real-time prediction of weld penetration depth using deep learning and sensor fusion—offering a promising alternative to conventional destructive or radiographic testing methods.

## Figures and Tables

**Table 1:** Chemical composition of base material (wt.%).

	C	Mn	Si	P	S
Steel (780DP)	0.12	2.6	0.6	0.3	0.003

**Table 2:** Mechanical properties of base material.

	Tensile Strength (MPa)	Elongation (%)
Steel (780DP)	Min. 780	14

**Table 3:** Laser welding parameters.

**Laser power (W)**	1429~2750
**Welding speed (m/min)**	3, 4, 5, 6, 7
**Laser beam diameter (mm)**	0.27
**Focal length (mm)**	200

**Table 4:** Training results according to deep learning models.

**Model**	**Transfer learning; uni-sensor 100 Hz**				**Transfer learning with fine-tuning;** **uni-sensor 100 Hz**			
	**M**	**R**	**E**	**X**	**M**	**R**	**E**	**X**
MAE (mm)	0.007	0.007	0.007	0.014	0.009	0.022	0.010	0.018
R^2^	0.999	0.999	0.999	0.998	0.999	0.998	0.998	0.998
**Model**	**Transfer learning with fine-tuning;** **uni-sensor 500 Hz**				**Transfer learning with fine-tuning;** **multi-sensor 100 Hz**			
	**M**	**R**	**E**	**X**	**M**	**R**	**E**	**X**
MAE (mm)	0.009	0.009	0.007	0.008	0.010	0.016	0.017	0.013
R^2^	0.999	0.999	0.998	0.999	0.999	0.999	0.998	0.999

M: MobileNetV2; R: ResNet50V2; E: EfficientNetB3; X: Xception.

**Table 5:** Validation results according to deep learning models.

**Model**	**Transfer learning; uni-sensor 100 Hz**				**Transfer learning with fine-tuning;** **uni-sensor 100 Hz**			
	**M**	**R**	**E**	**X**	**M**	**R**	**E**	**X**
MAE (mm)	0.175	0.150	0.142	0.195	0.159	0.113	0.127	0.159
R^2^	0.614	0.705	0.677	0.510	0.668	0.834	0.771	0.668
**Model**	**Transfer learning with fine-tuning;** **uni-sensor 500 Hz**				**Transfer learning with fine-tuning;** **multi-sensor 100 Hz**			
	**M**	**R**	**E**	**X**	**M**	**R**	**E**	**X**
MAE (mm)	0.140	0.097	0.052	0.078	0.085	0.159	0.063	0.078
R^2^	0.739	0.871	0.945	0.915	0.902	0.668	0.943	0.917

M: MobileNetV2; R: ResNet50V2; E: EfficientNetB3; X: Xception.

**Table 6:** Test results according to deep learning models.

**Model**	**Transfer learning; uni-sensor 100 Hz**				**Transfer learning with fine-tuning;** **uni-sensor 100 Hz**			
	**M**	**R**	**E**	**X**	**M**	**R**	**E**	**X**
MAE (mm)	0.180	0.180	0.152	0.196	0.159	0.113	0.128	0.159
R^2^	0.564	0.564	0.681	0.502	0.688	0.837	0.800	0.688
**Model**	**Transfer learning with fine-tuning;** **uni-sensor 500 Hz**				**Transfer learning with fine-tuning;** **multi-sensor 100 Hz**			
	**M**	**R**	**E**	**X**	**M**	**R**	**E**	**X**
MAE (mm)	0.165	0.095	0.049	0.077	0.086	0.085	0.058	0.073
R^2^	0.617	0.875	0.951	0.915	0.901	0.900	0.956	0.927

M: MobileNetV2; R: ResNet50V2; E: EfficientNetB3; X: Xception.

## Data Availability

The original contributions presented in this study are included in the article. Further inquiries can be directed to the corresponding author.
